# Supplementary material for: Nanostructured Hybrids Based on Tantalum Bromide Octahedral Clusters and Graphene Oxide for Photocatalytic Hydrogen Evolution
Source: Nanomaterials (Basel). 2022 Oct 18;12(20):3647. doi: 10.3390/nano12203647 (PMC9611948; doi:10.3390/nano12203647)
Supplement: Supplementary file 1 [file nanomaterials-12-03647-s001.zip › nanomaterials-1971368-supplementary.pdf]

Supporting Information

Nanostructured hybrids based on Tantalum Bromide Octahedral Clusters and Graphene Oxide for Photocatalytic Hydrogen Evolution

John Sebastián Hernández [1], Maxim Shamshurin [2], Marta Puche [1], Maxim N. Sokolov [2], and Marta Feliz [1]

[1] Instituto de Tecnología Química, Universitat Politècnica de València- Consejo Superior de Investigaciones Científicas (UPV-CSIC), Avd. de los Naranjos s/n, 46022 Valencia, Spain
[2] Nikolaev Institute of Inorganic Chemistry SB RAS, 3 Akad. Lavrentiev Ave., Novosibirsk, 630090, Russian Federation.

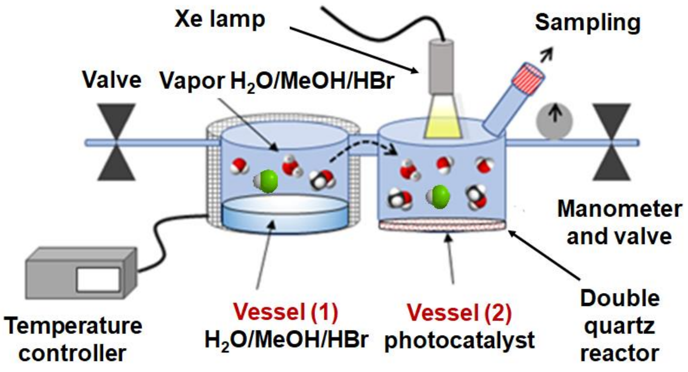

Figure S1. Experimental setup for photocatalytic hydrogen production in vapor phase conditions.

Table S1. Amount of H_2 produced in control tests done under standard photocatalytic conditions.

Test	Control conditions				H_2 produced (μmol)
	Photocatalyst ($\{Ta_6Br^i_{12}\}$@GO-20L)	Light	MeOH	HBr	
1	No	Yes	Yes	Yes	0.08
2	Yes	No	Yes	Yes	0.08
3	Yes	Yes	No	Yes	0.14
4	Yes	Yes	Yes	No	0.11

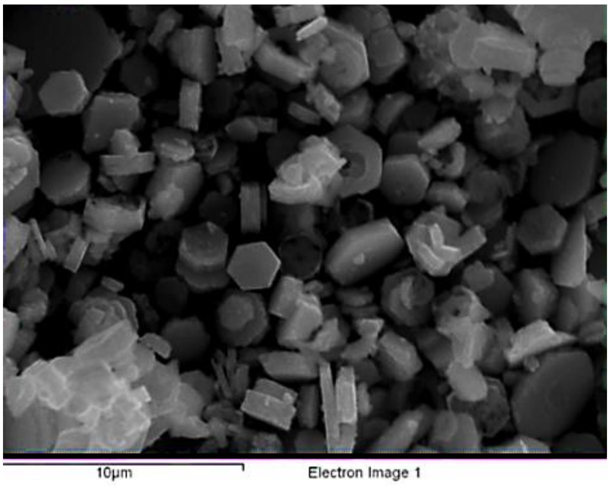

Figure S2. SEM image of $[\{Ta_6Br^i_{12}\}Br^a_2(H_2O)^a_4]\cdot 4H_2O$.

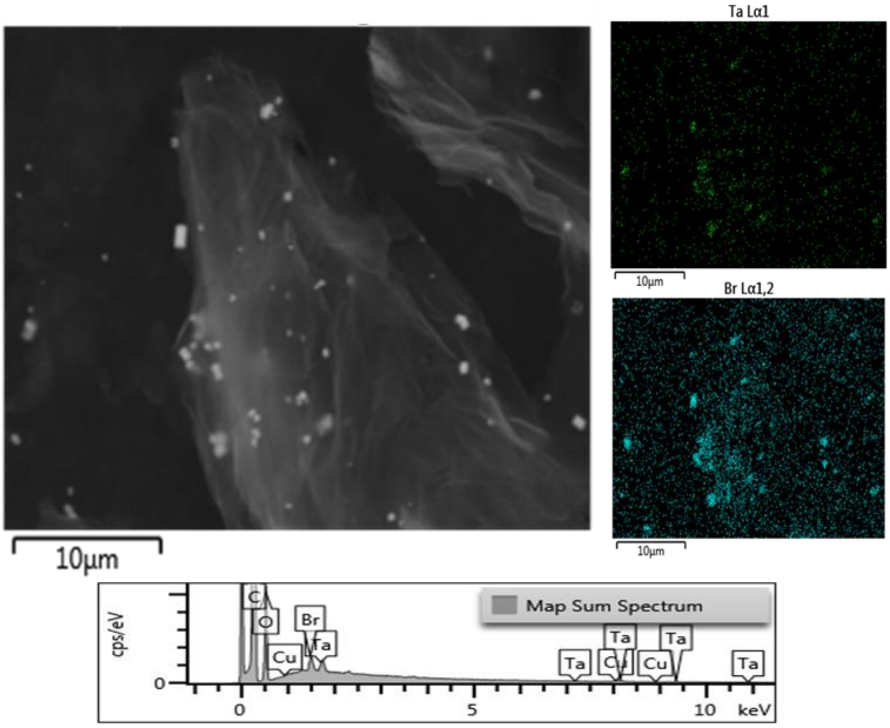

Figure S3. EDS analysis of $\{Ta_6Br^i{}_{12}\}$@GO-20L showing peaks of the mass percentage of bromine and tantalum. The Cu signal appear is not significant because the calculated concentration of this metal was below the quantification limit of the technique (< 0.05% w/w).

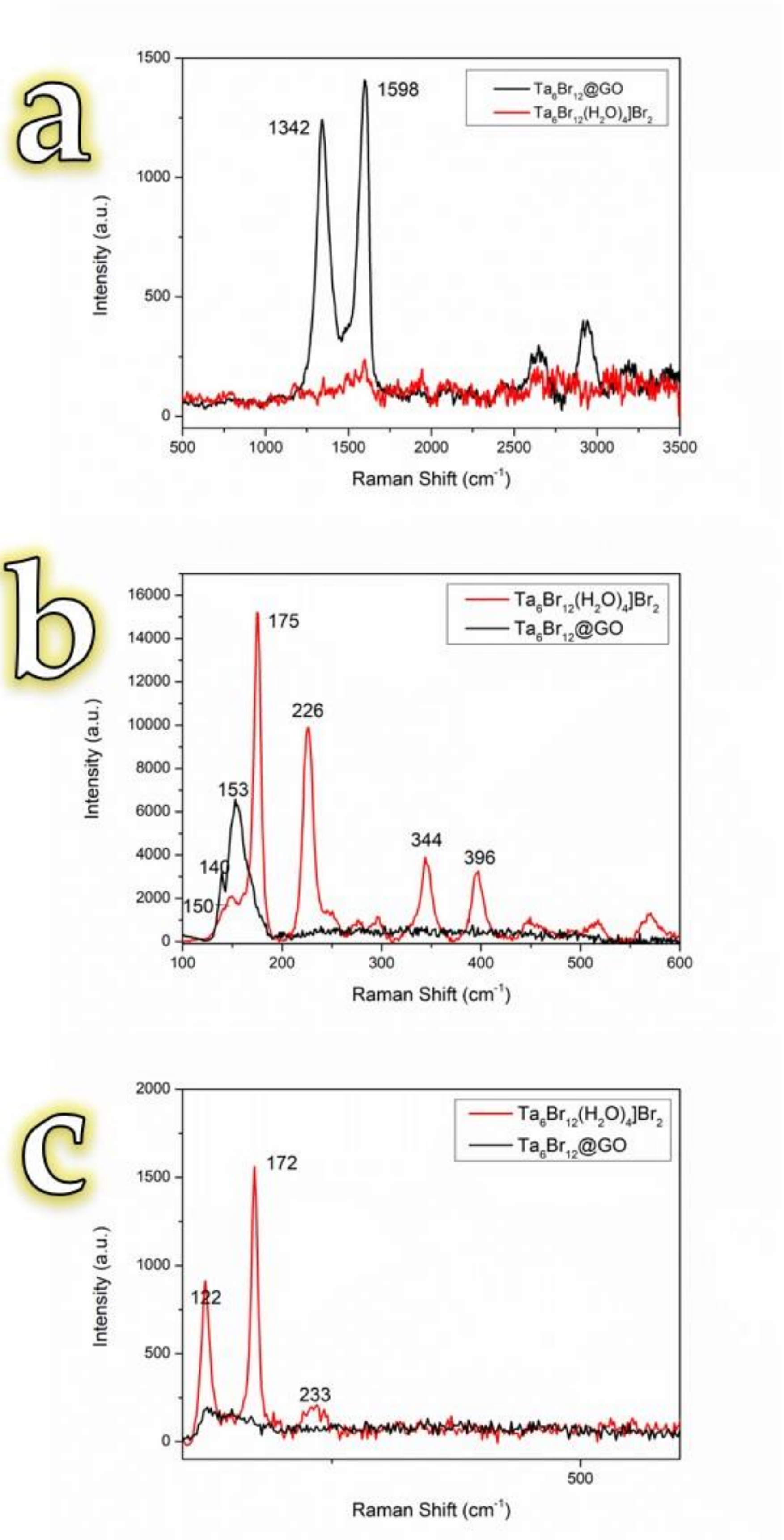

Figure S4. Raman spectra of $\{Ta_6Br^i_{12}\}$@GO-20L and $[\{Ta_6Br^i_{12}\}Br^a_2(H_2O)^a_4]\cdot 4H_2O$ materials recorded at 785 nm (a) and (b), and at 514 nm (c).

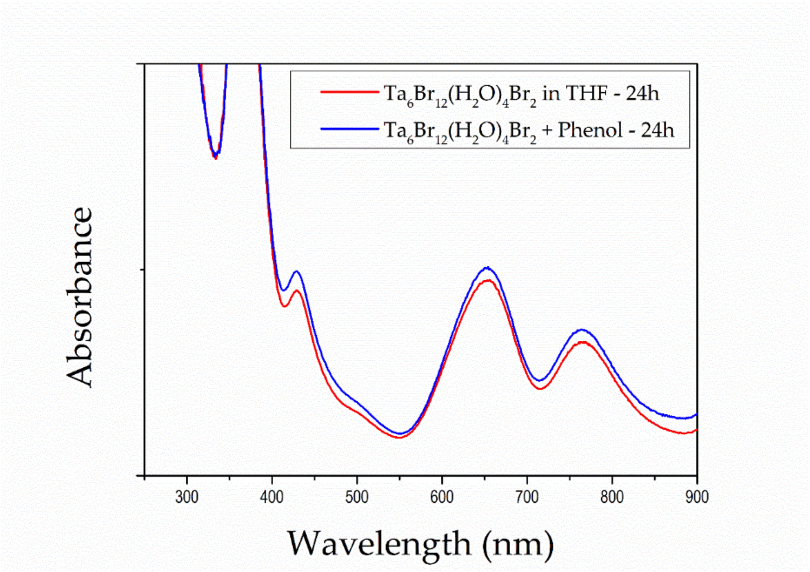

Figure S5. UV-Vis spectra of the (i) and (ii) solutions.

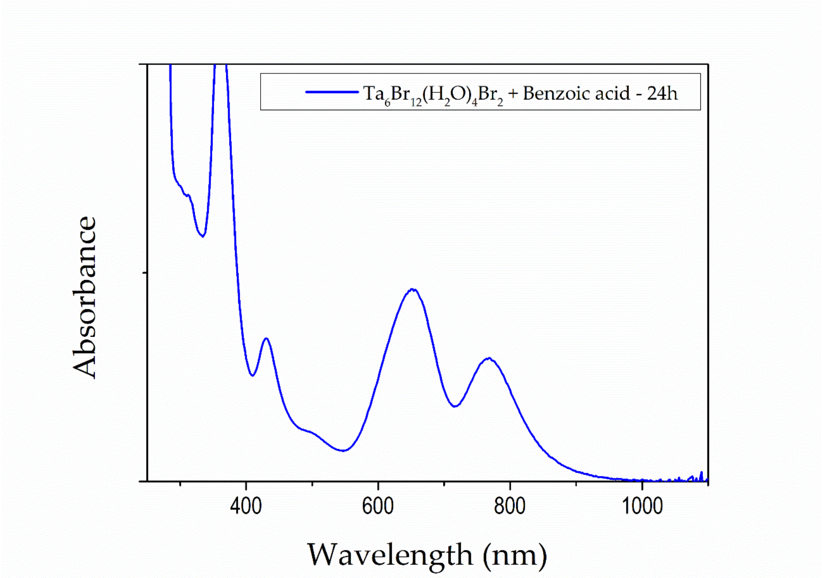

Figure S6. UV-Vis spectra of the (iii) solution.

Table S2. Optimal catalytic activities of $\{Ta_6Br^i_{12}\}$@GO-20L compared to octahedral molybdenum cluster-based photocatalysts and selected tantalum photocatalysts for H_2 production from water.[a]

Photocatalyst	Sacrificial agent	Co-catalyst /Photosensitizer	Activity (H_2 production, $\mu mol \cdot g_{cat}^{-1} \cdot h^{-1}$)	Ref.
**$\{Ta_6Br^i_{12}\}$@GO-20L**	methanol	None	4 [b]	This work
$(TBA)_2Mo_6I^i_8$@GO	methanol	None	3 [b]	[12]
$(TBA)_2[Mo_6I^i_8(O_2CCH_3)^a_6]$	methanol	None	7 [b]	[12]
$(TBA)_2Mo_6Br^i_8$@GO	methanol	None	2 [c]	[35]
$LiTaO_3$	None	None	6 [c]	[36]
$NaTaO_3$	None	None	4 [c]	[36]
$MgTa_2O_6$	None	None	5 [c]	[36]
$BaTa_2O_6$ (Hexagonal phase)	None	None	7 [c]	[36]
Ta_2O_5	methanol	H_2PtCl_6	8 [c]	[58]
Tantalum oxynitride	methanol	Pt (3% wt)	5 [c]	[65]
Ta_3N_5 nanoparticles	methanol	Pt (3% wt)	13 [c]	[66]
Ta_2O_5@NRGO	tetraethanolamine	None	6500 [c]	[67]

[a] Abbreviations: NRGO = nitrogen-doped reduced graphene oxide; [b] Results obtained from aqueous mixtures in vapor phase; [c] Results obtained from aqueous mixtures in liquid phase;
